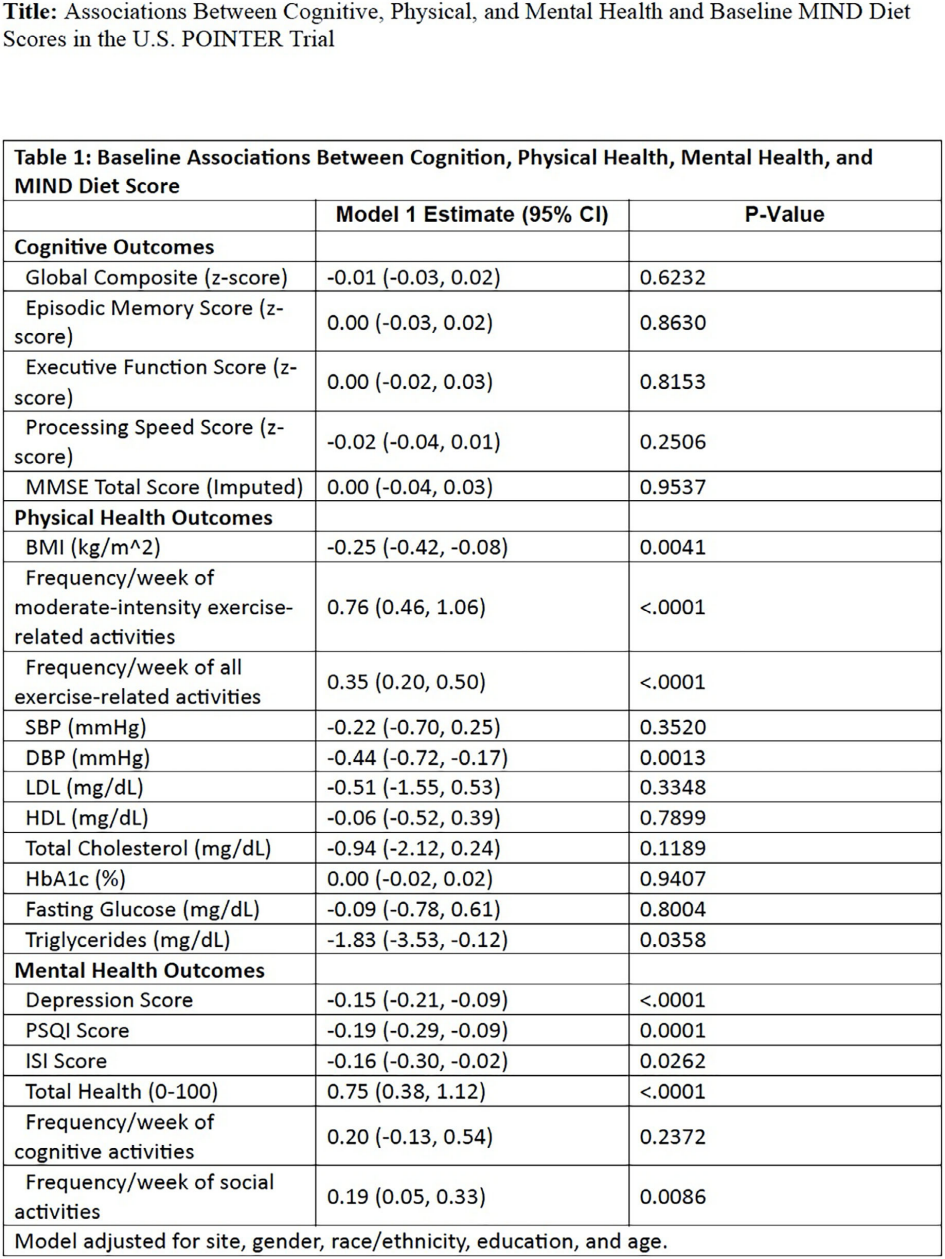# Associations Between Cognitive, Physical, and Mental Health and Baseline MIND Diet Scores in the U.S. POINTER Trial

**DOI:** 10.1002/alz.089812

**Published:** 2025-01-09

**Authors:** Jennifer Ventrelle, Jeffrey A. Katula, Christy C Tangney, Katelyn R Garcia, Kristin R Krueger, Sarah Graef, Sharon Wilmoth, Allison Heinrich, Desiree Lopez, Laura D Baker

**Affiliations:** ^1^ Rush University Medical Center, Chicago, IL USA; ^2^ Wake Forest University, Winston Salem, NC USA; ^3^ Rush University, Chicago, IL USA; ^4^ Wake Forest University School of Medicine, Winston‐Salem, NC USA; ^5^ Rush Institute for Healthy Aging, Chicago, IL USA; ^6^ Wake Forest University School of Medicine, Winston Salem, NC USA; ^7^ Wake Forest University, Winston‐Salem, NC USA

## Abstract

**Background:**

The Mediterranean‐Dash Intervention for Neurodegenerative Delay (MIND) diet, featuring emphasis on green leafy and other colorful vegetables, berries, unsaturated fats, fish, and whole grains is a major component of the U.S. POINTER multi‐domain lifestyle intervention. The purpose of this study is to examine the associations among MIND diet screener scores, and measures of cognitive function, and physical and mental health at baseline.

**Method:**

U.S. POINTER is a randomized controlled trial of two multidomain lifestyle interventions. (n = 2112; mean age = 68.18±5.15y). Cognitive function measures included global, episodic memory, executive function, and processing speed, derived from the U.S. POINTER modified Neuropsychological Test Battery. Physical and mental health were assessed using measures of BMI, hypertension, diabetes, dyslipidemia, physical activity, the Geriatric Depression Scale, the Pittsburgh Sleep Quality Index (PSQI) and symptoms of psychological distress. Pearson correlations were used to estimate bivariate associations among variables. Generalized linear regression models were used to examine associations while controlling for demographic variables.

**Result:**

Baseline MIND diet screener scores were inversely associated with BMI (r = ‐0.09, p<.0001), diastolic blood pressure (DBP) (r = ‐0.10, p<.0001), depression (r = ‐0.12, p<.0001), and PSQI (r = ‐0.09, p<0.0001). MIND diet scores were positively associated with frequency of moderate‐intensity exercise (r = 0.10, p<.000) and total health score (r = 0.09, p<.0001). Linear models revealed that after controlling for demographics, MIND diet scores remained associated with BMI, DBP, frequency of exercise, depression, and sleep quality and total health (See Table 1). MIND diet scores were not associated with measures of cognition.

**Conclusion:**

In U.S. POINTER, MIND diet scores were not associated with cognition; however, those with higher MIND diet scores had lower BMI, DBP depression and PSQI scores, as well as higher frequency of moderate‐intensity exercise, higher self‐reported health.